# Building trust through collaboration: a mixed-methods evaluation of San Francisco’s Pregnancy Village model of cross-sector care delivery

**DOI:** 10.1186/s13690-025-01808-9

**Published:** 2025-12-18

**Authors:** Osamuedeme J. Odiase, April J. Bell, Alison M. El Ayadi, Catherine Ravikumar, Kattia Suarez Vargas, KaSelah Crockett, Malini A. Nijagal, Patience A. Afulani

**Affiliations:** 1 Department of Obstetrics, Gynecology, & Reproductive Sciences, University of California, San Francisco, CA USA; 2 Department of Family and Community Medicine, University of California, San Francisco, CA USA; 3 Department of Epidemiology and Biostatistics, University of California, San Francisco, CA USA; 4 Department of Neurological Surgery, Weill Institute for Sciences, University of California, San Francisco, CA USA; 5 Institute for Health and Aging, University of California, San Francisco, CA USA; 6 Compass & Keys, Oakland, CA USA; 7 Pop-Up Village, Oakland, CA USA

**Keywords:** Trust, Medical mistrust, Community-institution partnership, Health system, Community-based organizations, Pregnancy, Perinatal health, Birth equity, Health equity, Program evaluation

## Abstract

**Background:**

Historical injustices, systemic racism, unequal healthcare access, and provider bias have fostered mistrust in healthcare institutions. Cross-sector collaborations between healthcare institutions and community-based organizations (CBOs), such as San Francisco’s Pregnancy Village (PV) model, could potentially build institutional trust within minoritized communities. This study primarily aimed to examine trust in PV, with secondary aims exploring participant perceptions of trust in the health system and CBOs, including their views on the health system’s involvement in PV.

**Methods:**

Between July 2021 and June 2022, we conducted a convergent, mixed-methods study involving 116 survey participants (57 pregnant/postpartum individuals and 59 family members) and 18 semi-structured interviews (13 pregnant/postpartum people and five family members). Trust was assessed quantitatively using a seven-item scale (scores standardized to 0-100) adapted from the Public Healthcare System Trust Scale and qualitatively with open-ended questions. We performed univariate, bivariate, and multivariate analyses of the quantitative data and thematic analyses of the qualitative data.

**Results:**

The mean trust in PV score was 85.9/100 (SD = 18.9), with lower trust among Latine participants (*β* = -12.2, 95% CI: -21.6, -2.9), those with prior preterm birth (*β* = -11.0, 95% CI: -20.5, -1.4), and those experiencing food insecurity (*β* = -12.4, 95% CI: -21.0, -3.8). Qualitative findings revealed that trust in both the health system and CBOs was shaped by receipt of person-centered care. Trust in CBOs was attributed to their focus on holistic care, relatability, and responsiveness to community needs. Distrust in the health system was shaped by experiences of racism and neglect. Participants held mixed views on the health system’s role in PV; some highlighted its ability to meet community needs, while others voiced skepticism due to ongoing structural racism and inequities in care.

**Conclusions:**

Participants perceived PV as trustworthy, with mixed views of the health system, generally positive perceptions of CBOs, and overall support for the health system’s involvement in PV despite lingering concerns regarding structural racism. These findings underscore PV’s unique role in bridging sectors and highlight that sustained, community-guided collaboration is essential to building trust and advancing more equitable cross-sector care.

**Supplementary Information:**

The online version contains supplementary material available at 10.1186/s13690-025-01808-9.

**Table:** 

Text box 1. Contributions to the literature
This study demonstrates that the Pregnancy Village, a cross-sector model of perinatal care, fosters high participant trust, shaped by race, language, preterm birth history, and food insecurity, providing evidence for the necessity of trauma-informed, culturally responsive approaches to address trust disparities in perinatal care.
Highlights greater trust in community-based organizations over health systems, linked to holistic, relatable, and community-responsive care, offering critical insights into effective care delivery approaches
Illuminates the impact of historical and ongoing racism on trust in healthcare, underscoring the need for systemic change
Advances understanding of how cross-sector collaborations can bridge historical divides between marginalized communities and healthcare institutions

## Background

Historical injustices, such as the Tuskegee Syphilis Study, forced sterilization under state eugenics programs, and medical exploitation of Black women, have fostered an entrenched mistrust of healthcare among Black individuals [1–4]. These injustices persist today through structural, systemic, and interpersonal racism, leading to unequal healthcare access and quality, which exacerbate disparities in maternal health [5–8] and reinforce a cycle of negative engagement with healthcare systems [9]. Community-based organizations (CBOs)—non-profit, non-governmental organizations committed to addressing community needs [10]—have emerged as pivotal stakeholders within the health ecosystem, which encompasses healthcare systems, CBOs, and public health and government agencies. Often characterized as part of the “third sector,” CBOs occupy a unique intermediary space, filling the gap between state-provided social services and traditional healthcare systems [11, 12].

CBOs play a vital role in Black communities, often acting as first responders in efforts to combat disparities in maternal and child health through services such as doula support, community health workers, and referrals to other social services, as well as education, housing, and economic empowerment programs [13, 14]. For instance, CBOs like SisterWeb provide free doula support and advocacy for Black families [15]; the Homeless Prenatal Program provides housing support and other services to low-income families [16]; and Rafiki Coalition for Health and Wellness provides a safe space for therapy, massages, chiropractic services, and other community resources in San Francisco [17]. Over time, CBOs have expanded their focus from immediate service provision to advocating for systemic change, social justice, and health equity [18–20].

Collaboration between CBOs and health systems is growing, with both entities recognizing a greater collective impact [21]. Health system leaders are increasingly involving CBOs in the development of health programs, services, and interventions [22–27]. This enhances the cultural relevance, acceptability, and effectiveness of health interventions [22, 28, 29], thereby contributing to increased institutional trust [21, 30]. However, these cross-sector collaborations face challenges like power imbalances, with health systems often dominating decision-making [31–33], and CBOs frequently expected to shoulder the dual responsibility of serving the community and building trust, often devoid of sustained health system-level reforms or institutional support [34]. These structural and relational barriers collectively signal the need for a substantive re-envisioning of how these sectors work together to address community needs and build trust.

Innovative, cross-sector, community-driven models have the potential to build trust in Black and minoritized communities [35]. One such model is the “Pregnancy Village” (PV) model—a cross-sector collaboration, providing a “one-stop shop” of comprehensive wellness services in a safe and uplifting environment for those who face the starkest inequities: Black-identifying pregnant and postpartum individuals. Following a three-year planning phase in collaboration with the creators of the “Pop-Up Village” event model, Designing Justice + Designing Spaces, the first iteration of the Pregnancy Village—the “SF Family & Pregnancy Pop-Up Village” (subsequently referred to as the “Pregnancy Village” (PV) for brevity)—was launched as a monthly event series from July 2021 to June 2024 [36].

Recognizing that trust is vital for sustaining community sociomedical interventions like PV, the PV model positions trust as a core organizing principle to enable relationship-building, foster meaningful community engagement, and ultimately support the model’s overall effectiveness. Therefore, as part of the evaluation of PV, we sought to assess participants’ perceptions of PV’s trustworthiness and key predictors. Additionally, we explored participants’ perceptions of trust in CBOs and the health system, as well as their perceptions of the health system’s role in PV. This paper presents findings from the formative phase—the first nine months of PV implementation (July 2021-June 2022)—as part of ongoing community engagement efforts to refine the PV model.

## Methods

### Setting

The Pregnancy Village (PV) holds events in San Francisco, California’s Bayview district, a neighborhood of 35,000 residents, known as the city’s “most isolated” [37], with significant inequities in care access, experience, and outcomes. Most birthing individuals in Bayview are Medicaid-insured (62%), and 93% are from racial or ethnic minority groups [38]. Bayview residents also experience markedly lower rates of timely prenatal care, higher rates of preterm birth, and low birth weight [39]. The Bayview has industrial, commercial, and residential zones, but declining industry has led to reduced infrastructure investment [40]. PV events were held near a major roadway to leverage proximity to public transportation and CBOs, aiming to build trust and foster community engagement [40].

### Intervention

**Fig. 1:**
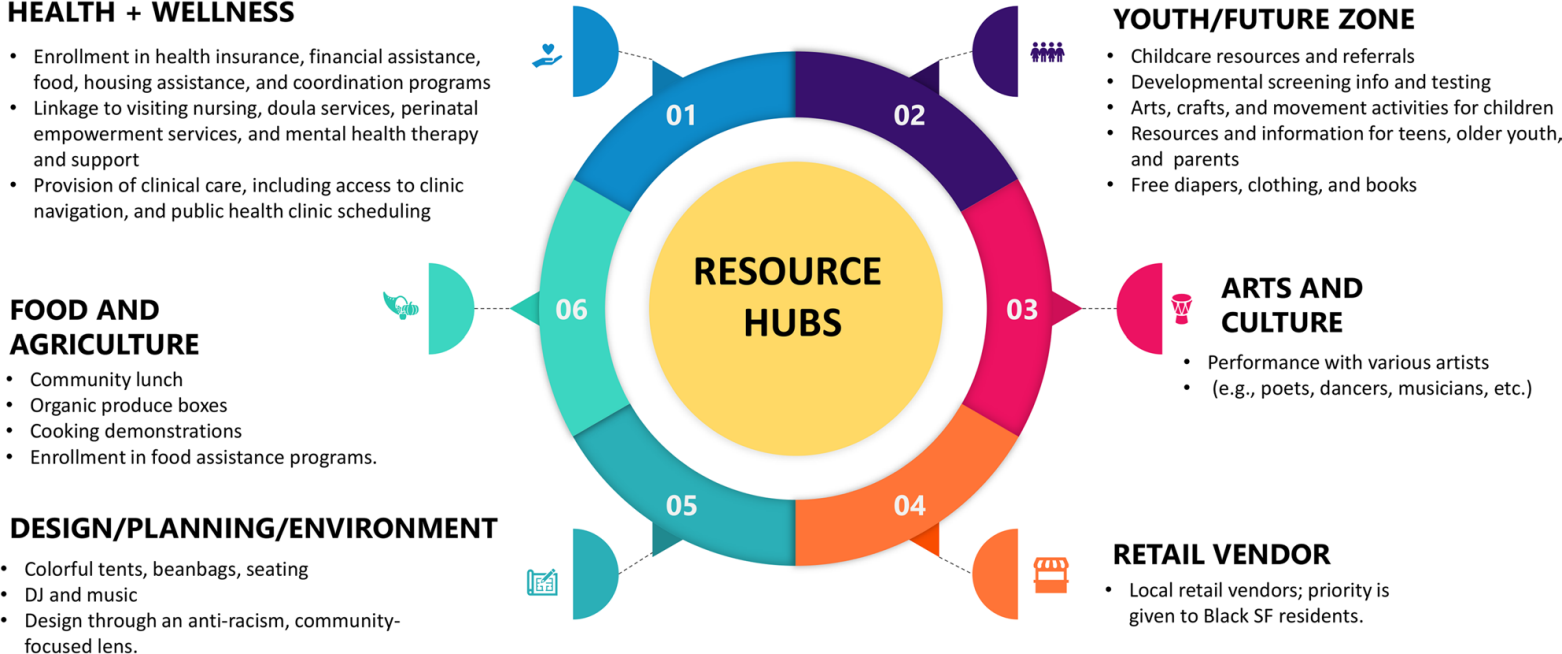
The Family and Pregnancy Pop-Up Village summary of provided services

PV is a model for cross-sector collaboration designed to address perinatal inequities by providing a one-stop-shop for resources in a celebratory and uplifting environment for Black pregnant individuals and their families. This care delivery model is rooted in person-centered and anti-racism principles and promotes sustainable community-institutional partnerships. It also features a real-time community feedback system that employs innovative data collection methods like vision boards with prompt-based sticky notes and a ‘Food-for-Thought’ social initiative, where community members share their experience and feedback in exchange for a bag of fresh produce to ensure responsive model iteration based on community’s stated needs [41]. PV features six distinct resource hubs (neighborhoods), each representing essential components needed to support comprehensive wellness within communities [36] (Fig. 1). The event design features vibrant colors, shaded areas, ground treatments, and varied seating arrangements to create an inviting space. Further details about the implementation of PV can be found elsewhere [36].

### Study design

We conducted a convergent mixed-methods, community-engaged evaluation, in which quantitative and qualitative data were collected concurrently through surveys and in-depth reviews, analyzed independently, and then integrated to interpret the findings. Integration was further supported by selecting a subset of participants for the in-depth interviews and developing the interview guide to explore topics introduced in the survey. This paper presents data related to trust in PV and participant perceptions of trust in the health system and CBOs, including their views on the health system’s involvement in PV.

### Sample

We recruited a convenience sample of pregnant and postpartum individuals, along with family members participating in the first nine monthly PV events between July 2021 and June 2022. The inclusion criteria were: (1) individuals must be at least 15 years old if pregnant or postpartum, or 18 years old if they were family members; (2) attendance at a minimum of one PV event; and (3) the ability to speak either English or Spanish. We focused on recruiting Black and other minoritized pregnant or postpartum individuals and their families, given that they were the focus population for PV, with a goal of 120 participants (10–15 individuals from each event) for the surveys. We then purposively sampled a subset of 18 participants (13 pregnant and postpartum individuals and 5 family members) for semi-structured in-depth interviews, guided by the principle of maximum variation to capture a wide range of experiences, including race/ethnicity, language, pregnancy status, and age (Additional file 1) [42]. Interviews continued until no additional themes emerged, indicating that thematic saturation had been achieved [43].

### Recruitment and data collection

Eligible participants were recruited during PV registration, where study team members provided details about the study and obtained verbal informed consent. Those who consented were given the option to complete a survey onsite using a study tablet or later on their own device via a QR code. Most participants completed the survey onsite and were compensated with a $20 gift card for their participation. Those who attended multiple PV events were permitted to complete the survey more than once. In addition, three to four participants from each PV event were approached during the events or contacted by phone to invite them for one-on-one in-depth interviews. Interviews were held within four weeks of attending a PV event, scheduled at participants’ convenience, and conducted via Zoom in English or Spanish. The semi-structured interview guide used included questions to explore: (1) trust in the health system; (2) trust in CBOs; and (3) perceptions of the health system’s involvement in PV to help understand trust in PV. Interviews lasted 30–60 min and were conducted by trained qualitative researchers (OJO and KV). Interview participants were compensated an additional $20. With participants’ consent, the interviews were recorded, transcribed by a third-party transcriptionist, and verified by study team members (KV and JV). Field notes were taken during the interviews, and a standardized template aided in rapid analysis for model iteration and assessment of saturation [36]. These methods have been previously described elsewhere [40].

### Quantitative measures

*Trust in PV:* Trust in PV was evaluated using a 7-item scale adapted from the validated Public Healthcare System Trust Scale designed to assess trust in public healthcare systems [44] (See individual items in Additional file 2). Items most relevant to the PV context were selected with input from a community advisory board (CAB) and cognitive interviews with three pregnant and postpartum individuals from Bayview and the greater San Francisco Bay Area, to enhance content validity.

The adapted scale consisted of seven closed-ended questions, with responses provided on a 4-point Likert scale: 3 for “Strongly agree,” 2 for “Agree,” 1 for “Disagree,” and 0 for “Strongly disagree.” The responses were summed and then standardized on a scale of 0 to 100 by dividing the score by the maximum possible score and multiplying by 100, with higher scores indicating a higher level of trust. Missing data (2.6%) were imputed with the mean of the other responses within the measure. The internal reliability consistency for the trust scale within this sample was Cronbach’s α = 0.95.

*Covariates*: Participants self-reported sociodemographic characteristics, pregnancy and obstetric factors, and experiences of discrimination (See Table 1).

**Table 1:** Characteristics of pregnancy village study participants, *N* = 89

Participant Characteristics	Total (*N* = 89)		Currently pregnant/recently pregnant (*n* = 47)		Family (*n* = 42)	
	*N*	%	*n*	%	*n*	%
*Age*						
15–24	13	14.6	10	21.3	3	7.1
25–34	28	31.5	19	40.4	9	21.4
35–44	18	20.2	13	27.7	5	11.9
45 and older	19	21.4	1	2.1	18	42.9
Unknown	11	12.4	4	8.5	7	16.7
*Gender*						
Female	77	86.5	42	89.4	35	83.3
Male	6	6.7	0	0.0	6	14.3
Other/unknown/prefer not to answer	6	6.7	5	10.6	1	2.4
*Race/ethnicity*						
Black	32	36.0	13	27.7	19	45.2
Latine/Hispanic	34	38.2	20	42.6	14	33.3
Multiracial	17	19.1	10	21.3	7	16.7
Other/unknown/prefer not to answer	6	6.7	4	8.5	2	4.8
*Language*						
English	52	58.4	26	55.3	26	61.9
Spanish	29	32.6	16	34.0	13	31.0
Unknown/other/prefer not to answer	8	9.0	5	10.6	3	7.1
*English proficiency*						
Very well or well	63	70.8	34	72.3	29	69.1
With difficulty	16	18.0	8	17.0	8	19.1
Unknown/prefer not to answer	10	11.2	5	10.6	5	11.9
*Parity*						
No births	17	19.1	12	25.5	5	11.9
1–2 births	39	43.8	23	48.9	16	38.1
3 or more births	25	28.1	11	23.4	14	33.3
Unknown/not applicable/prefer not to answer	8	9.0	1	2.1	7	16.7
*Prenatal care*						
No prenatal care during current/recent pregnancy	3	3.4	3	6.4	0	0.0
Received prenatal care during current/recent pregnancy	39	43.8	39	83.0	0	0.0
Unknown/not applicable/prefer not to answer	47	52.8	5	10.6	42	100.0
*History of preterm birth*						
No preterm births	59	66.3	38	80.9	21	50
At least 1 preterm birth	21	23.6	7	14.9	14	33.3
Unknown/prefer not to answer/not applicable	9	10.1	2	4.3	7	16.7
*History of pregnancy loss*						
No prior pregnancy loss	50	56.2	26	55.3	24	57.1
Pregnancy loss	26	29.2	17	36.2	9	21.4
Unknown/prefer not to answer/not applicable	13	14.6	4	8.51	9	21.4
*Education*						
Less than a high school degree	17	19.1	6	12.8	11	26.2
High school graduate, GED or equivalent	23	25.8	15	31.9	8	19.1
Some college, junior college or vocational school	22	24.7	10	21.3	12	28.6
College graduate or higher	19	21.4	11	23.4	8	19.1
Unknown/prefer not to answer	8	9.0	5	10.6	3	7.1
*Employment*						
No	56	62.9	24	51.1	32	76.2
Yes, Full-time	14	15.7	10	21.3	4	9.5
Yes, Part-time	10	11.2	8	17.0	2	4.8
Unknown/prefer not to answer	9	10.1	5	10.6	4	9.5
*Income assistance*						
No	38	42.7	14	29.8	24	57.1
Yes	44	49.4	27	57.5	17	40.5
Unknown/prefer not to answer	7	7.9	6	12.8	1	2.38
*Residence*						
Bayview-Hunter’s Point	31	34.8	15	31.9	16	38.1
Other San Francisco	38	42.7	23	48.9	15	35.7
East Bay	10	11.2	6	12.8	4	9.5
Other/unknown/prefer not to answer	10	11.2	3	6.4	7	16.7
*Housing status*						
Homeless Shelter	10	11.2	6	12.8	4	9.5
Living with someone for free	5	5.6	3	6.4	2	4.8
No living place	1	1.1	0	0.0	1	2.4
Owns home or apartment	8	9.0	3	6.4	5	11.9
Public Housing	15	16.9	6	12.8	9	21.4
Renting home or apartment	39	43.8	23	48.9	16	38.1
Transitional or supportive housing	2	2.3	1	2.1	1	2.4
Other/unknown/prefer not to answer	9	10.1	5	10.6	4	9.5
*Social Support*						
Not at all	10	11.2	2	4.26	8	19.1
A little	9	10.1	7	14.9	2	4.8
Somewhat	20	22.5	11	23.4	9	21.4
Yes, definitely	48	53.9	26	55.3	22	52.3
Unknown/prefer not to answer	2	2.3	1	2.1	1	2.4
*Medical insurance status*						
Private insurance	15	16.9	10	21.3	5	11.9
Public insurance	58	65.2	30	63.8	28	66.7
No insurance	4	4.5	0	0.0	4	9.5
Unknown/prefer not to answer	12	13.5	7	14.9	5	11.9
*Food insecurity (Worried food would run out)*						
Often true	12	13.5	4	8.51	8	19.1
Sometimes true	22	24.7	14	29.8	8	19.1
Never true	40	44.9	18	38.3	22	52.4
Unknown/prefer not to answer	15	16.9	11	23.4	4	9.5
*Food insecurity (Insufficient funds)*						
Often true	11	12.4	5	10.6	6	14.3
Sometimes true	27	30.3	16	34.0	11	26.2
Never true	36	40.5	16	34.0	20	47.6
Unknown/prefer not to answer	15	16.9	10	21.3	5	11.9
*Relationship status*						
Partnered, living together	31	34.8	19	40.4	12	28.6
Partnered, not living together	15	16.9	9	19.2	6	14.3
Single	33	37.1	11	23.4	22	52.4
Other/unknown/prefer not to answer	10	11.2	8	17.0	2	4.8
*Everyday discrimination experience*						
Never	17	19.1	10	21.3	7	16.7
Rarely	19	21.4	12	25.5	7	16.7
Sometimes	34	38.2	16	34.0	18	42.9
Often	17	19.1	8	17.0	9	21.4
Unknown/prefer not to answer	2	2.3	1	2.1	1	2.4
*Prenatal care discrimination experience*						
Never	35	39.3	14	29.8	21	50.0
Rarely	17	19.1	12	25.5	5	11.9
Sometimes	23	25.8	17	36.2	6	14.3
Often	8	9.0	3	6.4	5	11.9
Unknown/prefer not to answer	6	6.7	1	2.1	5	11.9

### Quantitative analysis

The analytic sample includes 116 participants, excluding four participants deemed ineligible after reviewing their demographic data. Participants who attended several monthly PV events were permitted to fill out one survey for each event they attended. In total, fifteen individuals completed the survey multiple times: one participant completed it eight times, another completed it six times, two participants completed it three times, and eleven completed it twice. Overall, 89 unique individuals participated in the evaluation. We first examined trust scores and the covariates using descriptive statistics (means for continuous variables and percentages for categorical variables), followed by bivariate associations between trust scores and the covariates using ordinary least squares (OLS) linear regressions, adjusting standard errors to account for clustering by the number of events attended. Variables with p-values less than 0.05 were then included in the final multivariate model, which was refined by assessing collinearity and model fit. To account for clustering due to some participants providing multiple responses, we estimated linear mixed-effects models. We conducted sensitivity analyses by excluding missing data and duplicate responses (subsequent survey responses from the same participant). All analyses were conducted in STATA (version 14) [45].

### Qualitative analysis

We employed a thematic analysis approach [46]. The qualitative lead (OJO) developed an initial deductive codebook based on the interview guide. Six analysts (OJO, PD, KV, EK, HS, and KM) then independently coded the transcripts using Dedoose software [47]. Two transcripts were collaboratively coded, during which the codebook was refined by iteratively adding inductive codes to capture emerging concepts not outlined in the interview guide. To ensure inter-rater reliability, each of the remaining transcripts was independently coded by two analysts, with the qualitative lead reviewing the coding for consistency. Seven codes related to trust were synthesized into a thematic table with five categories: (1) Trust in the health system; (2) distrust in the health system; (3) trust in CBOs; (4) distrust in CBOs; and (5) perceptions of the health system’s involvement in PV.

## Results

### Quantitative results

#### Participant characteristics

Participants represented a diverse sample in terms of age, race and ethnicity, education, and socioeconomic status. A little over half were currently or recently pregnant (within the past year), with ages ranging from 17 to 76 years. 36% identified as Black and 38% as Latine. Educational attainment varied, with less than half having post-secondary education. Approximately two-thirds reported unemployment, and nearly half received income assistance; two-thirds had public health insurance. Notably, about one in four had experienced at least one preterm birth, and 29% experienced a pregnancy loss. A full description of sociodemographic and obstetric health characteristics of the participants is detailed in Table 1.

#### Trust in PV and associated factors

The sample’s standardized mean trust in PV score was 85.9 (*SD* = 18.9) overall, 83.5 (*SD* = 18.5) for pregnant and postpartum recipients, and 88.4 (*SD* = 19.1) among family members, (*p* = 0.170) (see Table 2). The mean trust score for Black participants was 91.5 (*SD* = 13.4), compared to a mean trust score of 81.9 (*SD* = 21.2) for participants from other racial and ethnic groups, (*p* = 0.0071).

**Table 2:** Distribution of standardized scale scores for trust in PV

	*N*	Score	SD	Minimum Score	Maximum Score
Combined Sample	113	85.9	18.9	0.0	100.0
Pregnant/postpartum	57	83.5	18.5	0.0	100.0
Family member	56	88.4	19.1	0.0	100.0
Black participants	47	91.5	13.4	66.7	100.0
Participants from all other racial/ethnic groups	66	81.9	21.2	0.0	100.0

*Abbreviations:* SD Standard deviation

Table 3 shows the bivariate results for trust scores from the main sample, with subgroup analyses (pregnant/postpartum individuals and family members) in Additional file 3. Compared to Black individuals, English speakers, and those proficient in English, Latine individuals (95% CI: −21.6, −2.9), Spanish speakers (95% CI: −21.9, −6.8), and those with limited English proficiency (95% CI: −27.7, −1.1), respectively, had significantly lower trust in PV. Also, participants aged 25 to 44, with at least three births, one preterm birth, and those who occasionally experienced food insecurity (worried food would run out) had lower trust in PV compared to those aged 15 to 24, no prior birth, no preterm birth history, and no food insecurity, respectively. Conversely, higher trust was observed among participants who did not disclose their gender, had some higher education, and did not disclose their social support status, compared to those identifying as female, high school graduates, and with social support, respectively. 

**Table 3:** Bivariate results for predictors of trust in PV scores, *N* = 116

Predictor variables		Cross Tabs		OLS Regression (Unadjusted)			
	*N (%)*	*Mean*	*SD*	*Coeff.*	*[95% CI]*		*P-value*
*Age*							
15–24 [Reference Group]	13 (11.5)	89.7	13.4	0.0			
25–34	31 (27.4)	80.5	15.6	−9.3	−16.3	−2.2	0.022
35–44	25 (22.1)	77.3	28.1	−12.4	−24.3	−0.5	0.044
45 and older	30 (26.6)	94.6	10.7	4.9	−5.3	15.0	0.254
Unknown	14 (12.4)	90.8	14.0	1.1	−10.4	12.5	0.808
*Gender*							
Female [Reference Group]	100 (88.5)	85.4	19.3	0.0			
Male	6 (5.3)	81.7	20.3	−3.7	−15.0	7.6	0.416
Other/unknown/prefer not to answer	7 (6.2)	95.9	4.3	10.5	3.5	17.5	0.014
*Race/ethnicity*							
Non-Hispanic Black [Reference Group]	47 (41.5)	91.5	13.4	0.0			
Hispanic/Latine	42 (37.1)	79.3	24.0	−12.2	−21.6	−2.9	0.022
Multiracial	18 (15.9)	83.6	15.0	−7.9	−19.7	3.9	0.136
Other/unknown/prefer not to answer	6 (5.3)	95.2	5.2	3.7	−2.2	9.7	0.155
*Language*							
English [Reference Group]	69 (61.1)	90.3	13.8	0.0			
Spanish	35 (30.9)	75.9	24.9	−14.4	−21.9	−6.8	0.006
Other/unknown/prefer not to answer	9 (8.0)	91.0	10.8	0.7	−14.5	16.0	0.900
*English proficiency*							
Very well or well [Reference Group]	82 (72.6)	89.7	14.0	0.0			
With difficulty	20 (17.7)	75.2	30.5	−14.4	−27.7	−1.1	0.039
Unknown/prefer not to answer	11 (9.7)	77.1	14.9	−12.6	−18.1	−7.2	0.003
Pregnancy status							
Family member [Reference Group]	56 (49.6)	88.4	19.1	0.0			
Pregnant or postpartum	57 (50.4)	83.5	18.5	−4.9	−19.0	9.3	0.392
*Parity*							
1–2 births [Reference group]	50 (44.3)	87.8	14.1	0.0			
No births	26 (23.0)	92.9	12.1	5.0	−8.3	18.4	0.353
3 or more births	28 (24.8)	75.9	27.0	−12.0	−23.4	−0.5	0.044
Unknown/prefer not to answer/not applicable	9 (7.9)	86.2	17.6	−1.6	−12.0	8.9	0.699
*History of preterm birth*							
No preterm births [Reference Group]	80 (70.8)	88.4	17.1	0.0			
At least 1 preterm birth	23 (20.4)	77.4	23.5	−11.0	−20.5	−1.4	0.033
Unknown/prefer not to answer/not applicable	10 (8.8)	85.2	16.9	−3.2	−16.1	9.7	0.534
*History of pregnancy loss*							
No prior pregnancy loss [Reference Group]	69 (61.0)	88.3	17.6	0.0			
Pregnancy loss	29 (25.7)	80.8	22.0	−7.5	−21.3	6.3	0.207
Unknown/prefer not to answer/not applicable	15 (13.3)	84.8	17.0	−3.5	−17.4	10.4	0.523
*Education*							
High school graduate, GED, or equivalent [Reference Group]	21 (18.6)	82.7	14.7	0.0			
Less than high school degree	30 (26.6)	81.9	24.5	−0.8	−19.0	17.3	0.904
Some college, junior college, or vocational school	32 (28.3)	93.0	12.5	10.3	0.5	20.2	0.044
College graduate and professional or graduate school	20 (17.7)	83.8	25.4	1.1	−7.9	10.2	0.751
Unknown/prefer not to answer	10 (8.8)	85.2	15.3	2.5	−5.9	11.0	0.451
*Employment status*							
Unemployed [Reference Group]	74 (65.5)	86.4	17.8	0.0			
Full-time	16 (14.2)	84.8	15.1	−1.6	−7.7	4.5	0.505
Part-time	12 (10.6)	84.1	29.4	−2.3	−25.9	21.3	0.801
Unknown/prefer not to answer	11 (9.7)	85.7	19.6	−0.7	−21.6	20.2	0.930
*Income assistance*							
No [Reference Group]	50 (44.3)	89.2	14.1	0.0			
Yes	55 (48.7)	82.7	22.5	−6.6	−17.9	4.8	0.185
Unknown/prefer not to answer	8 (7.0)	86.9	15.6	−2.3	−7.3	2.7	0.265
*Residence*							
Bayview-Hunter’s Point (San Francisco) [Reference Group]	39 (34.5)	86.1	20.4				
Other San Francisco	52 (46.1)	86.1	19.6	0.0	−17.1	17.1	1.000
East Bay	11 (9.7)	78.8	12.5	−7.3	−16.5	1.9	0.093
Other/unknown/prefer not to answer	11 (9.7)	91.3	14.1	5.3	−9.8	20.3	0.387
*Housing status*							
Rent home or apartment [Reference Group]	58 (51.3)	84.6	21.9	0.0			
Homeless shelter	12 (10.6)	81.3	15.5	−3.2	−20.1	13.7	0.626
Owns home or apartment	8 (7.1)	91.1	12.3	6.5	−3.7	16.7	0.152
Public housing	17 (15.1)	86.0	18.9	1.4	−18.0	20.9	0.848
Unknown/Other (e.g., living with someone for free, no living place, transitional housing etc.)	18 (15.9)	90.7	11.5	6.2	−5.4	17.7	0.212
*Social Support*							
Yes, definitely [Reference Group]	63 (55.7)	89.5	17.1	0.0			
No, not at all	15 (13.3)	82.2	15.6	−7.3	−18.2	3.7	0.139
A little	11 (9.7)	81.8	19.3	−7.7	−16.2	0.8	0.067
Somewhat	22 (19.5)	79.0	24.0	−10.5	−34.0	13.0	0.283
Unknown/prefer not to answer	2 (1.8)	97.6	3.4	8.1	0.7	15.5	0.038
*Medical Insurance*							
Public insurance (e.g., Medi-Cal, Medicaid, etc.) [Reference Group]	77 (68.1)	88.0	19.4	0.0			
Private or employer provided insurance	18 (15.9)	85.7	14.8	−2.3	−11.4	6.8	0.525
No insurance	5 (4.4)	76.2	18.1	−11.8	−25.2	1.6	0.071
Unknown/prefer not to answer	13 (11.5)	77.3	19.5	−10.7	−24.5	3.1	0.098
*Food insecurity (Worried food would run out)*							
Never true [Reference Group]	56 (49.6)	92.9	12.0	0.0			
Sometimes true	28 (24.7)	80.4	21.1	−12.4	−21.0	−3.8	0.016
Often true	13 (11.5)	75.5	27.5	−17.4	−40.6	5.8	0.106
Unknown/prefer not to answer	16 (14.2)	79.5	18.7	−13.4	−22.6	−4.2	0.016
*Food insecurity (Insufficient funds)*							
Never true [Reference Group]	50 (44.3)	91.6	14.0	0.0			
Sometimes true	32 (28.3)	81.5	21.1	−10.1	−23.8	3.7	0.111
Often true	13 (11.5)	74.7	26.9	−16.9	−39.3	5.5	0.105
Unknown/prefer not to answer	18 (15.9)	85.7	15.3	−5.9	−16.2	4.3	0.185
*Relationship status*							
Married/partnered, living together [Reference Group]	37 (32.7)	81.9	20.1	0.0			
Married/partnered, not living together	16 (14.2)	81.5	14.6	−0.3	−8.2	7.6	0.920
Single	49 (43.4)	90.0	18.6	8.1	−8.6	24.9	0.249
Other/unknown/prefer not to answer	11 (9.7)	87.4	19.5	5.6	−9.2	20.4	0.353
*Every day discrimination experience*							
Never [Reference Group]	20 (17.7)	85.2	16.4	0.0			
Rarely	30 (26.6)	84.6	26.0	−0.6	−13.5	12.2	0.897
Sometimes	44 (38.9)	86.3	16.8	1.0	−8.0	10.1	0.770
Often	19 (16.8)	87.7	12.8	2.5	−6.3	11.3	0.479
*Prenatal care discrimination experience*							
Never [Reference Group]	49 (43.3)	87.3	22.4	0.0			
Rarely	22 (19.5)	84.6	16.4	−2.6	−14.1	8.9	0.559
Sometimes	34 (30.1)	83.1	16.0	−4.2	−19.8	11.3	0.493
Often	8 (7.1)	92.9	11.7	5.6	−7.4	18.6	0.298

*Abbreviations*: 95% CI 95% confidence interval, SD Standard deviation

**Table 4:** Linear mixed-effects model of select predictors on trust in PV scores, ***N*** = 116

Predictor variables	Coeff.	[95% CI]		*P*-value
*Age*				
15–24 [Reference Group]	0.0			
25–34	−7.1	−19.0	4.8	0.240
35–44	−5.1	−17.0	6.8	0.402
45 and older	1.6	−10.6	13.8	0.795
Unknown	−0.1	−13.5	13.3	0.986
*Gender*				
Female [Reference Group]	0.0			
Male	4.3	−23.6	32.1	0.764
Other/unknown/prefer not to answer	6.8	−10.0	23.5	0.427
*Race/ethnicity*				
Non-Hispanic Black [Reference Group]	0.0			
Hispanic/Latine	13.0	−2.9	28.9	0.109
Multiracial	0.3	−9.4	10.1	0.945
Other/unknown/prefer not to answer	8.2	−10.4	26.8	0.390
*Language*				
English [Reference Group]	0.0			
Spanish	−11.5	−29.9	6.8	0.218
Other/unknown/prefer not to answer	−1.3	−16.8	14.3	0.871
*English proficiency*				
Very well or well [Reference Group]	0.0			
With difficulty	−9.0	−22.8	4.8	0.203
Unknown/prefer not to answer	−11.0	−26.6	4.5	0.163
*History of preterm birth*				
No preterm births [Reference Group]	0.0			
At least 1 preterm birth	−6.8	−14.9	1.2	0.096
Unknown/prefer not to answer/not applicable	−2.1	−27.3	23.2	0.873
*Education*				
High school graduate, GED, or equivalent [Reference Group]	0.0			
Less than high school degree	4.5	−5.5	14.4	0.380
Some college, junior college, or vocational school	4.4	−4.9	13.6	0.355
College graduate and professional or graduate school	−4.6	−14.7	5.6	0.379
Unknown/prefer not to answer	1.0	−11.8	13.9	0.875
*Food insecurity (Worried food would run out)*				
Never true [Reference Group]	0.0			
Sometimes true	−7.2	−15.9	1.5	0.104
Often true	−11.1	−22.7	0.4	0.058
Unknown/prefer not to answer	−8.5	−19.5	2.5	0.128
*Social Support*				
Yes, definitely [Reference Group]	0.0			
No, not at all	−2.3	−12.8	8.1	0.658
A little	−6.4	−19.5	6.7	0.340
Somewhat	−6.0	−14.0	2.0	0.144
Unknown/prefer not to answer	9.3	−33.4	52.0	0.669
*Mean dependent var*	85.883			
*Number of observations*	113			
*Prob > chi2*	0.000			
*SD dependent var*	18.876			
*Chi-square*	61.001			
*Akaike crit. (AIC)*	992.86			

Abbreviations: 95% CI: 95% confidence interval; SD: standard deviation

There were no statistically significant predictors in the final multivariate model (Table 4). Nevertheless, the direction of associations broadly aligned with the bivariate analyses, except for race and ethnicity. For example, participants with limited English proficiency (*β* = − 9.0, 95% CI: − 22.8, 4.8*)*, a history of preterm birth (*β* = − 6.8, 95% CI: − 14.9, 1.2*)*, or frequent food insecurity (*β* = − 11.1, 95% CI: − 22.7, 0.4) had lower trust in PV than English-proficient participants, those without a preterm birth history, and those not experiencing food insecurity, respectively. By contrast, Latine participants demonstrated higher trust in PV (*β* = 13.0, − 2.9, 28.9) than Black participants.

#### Sensitivity analyses

A sensitivity analysis, which excluded missing responses and limited participant engagement to their first PV encounter, yielded nearly identical standardized mean trust scores: 83.4 (SD = 19.8) for the main sample (*N* = 88), 82.7 (*SD* = 19.0) for pregnant and postpartum recipients (*n* = 47), and 84.3 (*SD* = 20.9) for family members (*n* = 41).

### Qualitative results

Qualitative findings are organized around three key areas: factors influencing trust in the health system, factors shaping trust in CBOs, and perceptions of health system involvement in PV. In general, participants expressed mixed levels of trust in the health system and high trust in CBOs. Most participants viewed the health system’s involvement in PV positively, though a few voiced skepticism about its role.

### Factors shaping trust in the health system

We identified two themes from participants’ descriptions of factors that shaped their trust in the health system: *Person-centered care and care satisfaction*, and *familiarity with the health system*.

#### Person-centered care and care satisfaction

Participants attributed their trust in the health system to the positive interactions they had with health care providers, which reflected core domains of person-centered care—care that is respectful of and responsive to individuals’ preferences, needs, and values [48]. This included provider responsiveness, such as provider friendliness, welcoming demeanor, and attentiveness, which reinforced trust.

*“I think that their X hospital’s healthcare providers – I mean*,* their healthcare*,* like nurses and doctors*,* are really nice and welcoming. And they make sure that I know that everything’s okay. And*,* if anything was wrong*,* you know*,* they would let me know. And they’re just really welcoming and nice.” —*Multiracial pregnant participant, less than 30 years old.

Trust was also tied to effective communication with participants, noting confidence in provider knowledge, comfort asking questions, responsiveness to their questions, and positive reassurances:

*“Maybe because*,* what they* [healthcare providers] *teach me*,* it turns out to be true […] And they are always positive. Like*,* even if I am in pain*,* “Oh*,* don’t worry. You’re going to be fine.” No. I am not fine. Especially at the delivery time. [laughs] It’s hard. […] Yeah. But you’re going to be fine after this – just hours after this pain. And then*,* you know when I forget the pain*,* I was like*,* “Wow. They were right.” [laughs] Yeah. It’s only over for the delivery.” —*Black Pregnant participant, between 30 and 44 years old.

In addition, autonomy in decision-making was tied to trust, with some participants noting that their trust in the health system stemmed from being empowered to make choices regarding their health, including changing physicians with whom they are dissatisfied:

*“Because they* [health system] *give you the opportunity to say “hey*,* I don’t like this*,* I don’t think this provider*,* meaning doctor*,* is showing my best interest*,* or* [acknowledging] *my thoughts on the way I want my healthcare to be.” They give you the right to change doctors*,* to change providers. So*,* I have to trust*,* you know*,* that this institution has given me the right to choose how my health is going to be. Because if they hadn’t given me the right to choose*,* I probably wouldn’t be pregnant now because my first OBGYN was telling me that I should have weight loss surgery before I tried to have another child […] I told her* [old provider] *my concerns*,* and you know*,* and after a couple weeks of me being with her […] I just immediately found myself another one*,* because I didn’t want to hear the negative thoughts that she was giving*,* and I didn’t want me to be stressed. With this new doctor*,* I haven’t been* [stressed]. *She listens to me*,* she calls me*,* her intern calls me and checks on me.” —*Black pregnant participant, between 30 and 44 years old.

Further, participants’ trust in the health system was linked to their perception of healthcare providers being in tune with their unique circumstances and making decisions based on that, in a way that increased their satisfaction with services received. For instance, one participant valued the exception made during her childbirth, allowing her to stay in the hospital despite not being adequately dilated at presentation. She appreciated this decision as it spared her from going home, where she lacked social support and feared suffering alone:

*“I was nervous* [of giving birth] *because I didn’t know what to expect*,* and I was by myself initially […] When I first went to the hospital*,* I wasn’t even dilated yet*,* and they were going to send me home. But when I was at home*,* I was having really bad contractions. And so*,* they made an exception. They’re like*,* “We’ll go ahead and keep you.” And I know they didn’t have to do that. Because I was zero centimeters dilated*,* even though I was having crazy contractions […] It was really hard to be here going through labor in my room. It was very intense. And I wanted to be close to the medical attention. And I didn’t want to go home and have to come right back. So*,* I was thankful that they made the exception for me*,* because I didn’t want to come home and suffer all by myself. It was really bad. But*,* I made it!” —*Latine postpartum participant, between 30 and 44 years old.

#### Familiarity with the health system

One participant attributed their trust in the health system to long-term engagement, highlighting the system’s consistent presence in her life:

*“I was born at X hospital*. *I’ve been there on and off for all my life. So*,* I really don’t know no other hospitals but X hospital […] I’ve been dealing with them*,* like I said*,* all my life. That’s the hospital I know.”* —Black family member, 45 years old and above.

### Factors shaping distrust in the health system

We identified three main themes from participants’ reports of factors shaping their distrust in the health system: *Experiences of racism and discrimination*, *health system unwilling to center Black women’s health*,* and lack of preventative care.*

#### Experiences of racism and discrimination

A few participants expressed distrust of the health system, citing personal or familial experiences of racism and discrimination, as well as the systemic racism faced by the Black community when accessing its services:

*“Because I know when I was born – like my siblings*,* most of us were born in X Hospital and my mom did experience a lot of racism*,* again that was the 80s*,* so hopefully I’m thinking things have changed. There is a caution*,* and I don’t want to speak for the whole Black community*,* but I know that I have and a lot of people I know who are Black*,* especially women*,* when it comes to like doctors and our trust to the system*,* hence why we don’t see people getting vaccinated.”* —Black pregnant participant, between 30 and 44 years old.

#### Health system unwilling to center black women’s health

A few participants expressed distrust in the health system, noting that it often neglects the health needs of Black women and fails to center them in the development of treatment plans. For instance, one participant highlighted the lack of adequate research on Black women’s health, which limits the system’s ability to effectively support their well-being:

*No. I don’t trust [laughs] hospitals*,* especially as a Black woman. I don’t trust them. They’re always telling me something about myself. You know*,* you go to get care for one thing*,* and then*,* they’re like*,* “Oh. Well*,* first of all*,* you need to lose weight.” Okay. It’s like*,* okay*,* beyond that*,* what can you do to help me? Like don’t center my weight as like the main focus of your health intervention. Because I just don’t feel like they have enough research being done that centers Black women to make me feel comfortable that they’re going to be centering me in the medical office. There’s just not enough research centered on Black women and their physical bodies and experiences and things like that. So*,* until that happens*,* I’m just not – [laughs] I don’t trust – I mean I’ll use it*,* but I don’t trust it.” —*Black postpartum participant, 45 years old and above.

#### Lack of preventative care

Participants criticized the system’s focus on crisis intervention rather than prevention, arguing that it is primarily designed to act only during crises or after conditions have worsened:

*“Because I don’t think the medical system is set up to help us prevent*,* but more so to intervene in a crisis. From my first pregnancy*,* the experience that I had with going to check-ups with X hospital and my homebirth team – the X hospital side was so negative because they would constantly talk about things that could happen and bad things*,* and the focus was so much on instilling fear.” —*Pregnant participant, less than 30 years old, race/ethnicity unknown.

### Factors shaping trust in community-based organizations

Most participants expressed a strong trust in CBOs to support their health and well-being, with some indicating greater trust in CBOs than in the health system. This was attributed to several factors, including CBOs’ provision of *person-centered care*, their *holistic care approach*, their *relatability*, and their *commitment to meet community’s needs*. Additionally, participants’ long-standing relationships with these organizations were key in fostering trust.

#### Provision of person-centered care

Participants highlighted that the nature of care they received from CBOs was a key factor in building their trust in these organizations. Their descriptions reflected several domains of person-centered care marked by responsive and compassionate staff who served as dedicated supporters and advocates. For example, one Black postpartum participant described how her doula, provided through a CBO, played a pivotal role, offering unwavering support throughout her pregnancy and passionately advocating for her during her hospital-based childbirth. This advocacy empowered the participant and facilitated a trauma-free birth experience by ensuring that the care provided aligned with the participant’s birthing preferences:

*“Because I did end up working with a doula. …. That*,* I think*,* was my saving grace because she just was like*,* “Girl*,* you don’t need to be induced.” […] “You got pregnant naturally […]*,* “That means your body was healthy and ready to have a baby. So*,* you can have this baby naturally” […] I got railroaded at the very end of my pregnancy by my healthcare providers trying to get me to agree to an induction. At one point*,* a nurse and my OB had double-teamed me while I was on my non-stress test*,* telling me about all of the risks of not getting induced. I* [would] *have agreed to be induced*,* 100%*,* had I not had the doula. It would have been the difference between me having a completely natural childbirth versus having a traumatic childbirth […] Instead*,* I worked with the doula. She had me laboring at home with my partner for most of the time […] When I got to the hospital*,* I was nine-and-a-half centimeters dilated. I was so far into my pregnancy* [close to birth] *that they couldn’t do any interventions. All they could do was barely put me on the labor and delivery table. And he came out in two pushes.” —*Black postpartum participant, 45 years old and above.

Participants also noted that CBOs excelled in clear, informed communication with individuals, families, and the community. They emphasized organizations' ability to be attentive, actively listen, and respond to the needs of the community.

*“I would trust them almost a 100% more than the hospital healthcare system*,* because they’re community-based and they are trained and attuned to listening to the community members. That’s the difference. It’s about how you listen. And the medical community – I mean*,* for all intents and purposes – has a God complex. They think they know everything. And they’re not great listeners.” —*Black postpartum participant, 45 years old and above.

One participant expressed appreciation for the respect shown by CBOs, particularly valuing the confidentiality of their discussions. They considered this trustworthiness a crucial aspect of their relationship with these organizations:

*“I think they* [CBOs] *are trusting. Because I feel like*,* whenever I ask them* [CBOs] *something or I tell them something*,* they really just keep it confidential*,* and it’s always just between me and them. I never feel like there’s someone else in the conversation.” —*Multiracial pregnant participant, less than 30 years old.

#### Holistic approach to care

Participants highlighted that CBOs earned their trust by prioritizing a holistic approach to care. These organizations focused on overall health and wellness, rather than relying on extensive medical interventions. Unlike the health system, CBOs were not constrained in their ability to provide comprehensive, personalized care:

“*They are not bound to the standards or rules that medical providers are necessarily bound to by the medical system. So*,* they can look at more holistic approaches and – yes*,* less medical intervention*,* but more medical prevention*.” *—*Pregnant participant, less than 30 years old, race/ethnicity unknown.

#### Relatability and approachability

Participants noted that CBOs were more relatable and approachable than the health system. Some compared their interaction with CBOs to a familial bond, which fostered a sense of comfort and encouraged open communication and problem-sharing:

*“Because*,* you know*,* for example*,* the hospital – X Hospital is like you are dealing with the doctors*,* nurses*,* and professionals. But*,* when we come to the communities* [CBOs], *I feel like*,* when we come to the communities* [CBOs], *you’re dealing with a mother like you […] And*,* with different perspective people*,* it’s even easier to approach. You know? Instead of dealing with upper – I feel like – let’s call them “upper class.” But this is like siblings*,* like sisters. […] So it feels easier to talk to them*,* you know*,* to share your problems*.*” —*Black Pregnant participant, between 30 and 44 years old.

#### Reliability and commitment

Participants expressed trust in CBOs for their reliability and consistent availability in meeting their needs:

*“I trust them because they’re always able to provide the help that I need. I guess with the two ones that I rely on*,* which are the material stuff—just like the diapers and wipes and the formula and my baby’s food.” —*Latine postpartum participant, less than 30 years old.

They also valued the organizations’ deep commitment to the community, noting how CBOs go above and beyond to meet individuals where they are and provide tailored support.

*“At this point*,* I do trust them. Because they* [CBOs] *could be doing something else with their time on a Saturday. They could be elsewhere with their family. But obviously*,* this might be something that they believe in*,* and they have a heart for it. And to give up your time*,* wow*,* that’s a lot. Time is something you can’t ever get back. You can’t get that back. So*,* that’s very special for me that they would donate their time like that.” —*Latine postpartum participant, between 30 and 44 years old.

#### Familiarity

Participants attributed their trust in CBOs to their familiarity with the organizations and the long-standing relationships they had built. This trust was further reinforced by their positive experiences with these organizations:

*“I’ve been going there for a long time*,* and they’ve been known to me*,* so I trust them.” —*Black family member, 45 years old and above.

*“I think so* [I trust them]. *Those* [CBOs] *are the places that I know. Because certainly*,* in the past*,* they helped me a little bit. They had the resources that I need.” —*Latine pregnant participant, between 30 and 44 years old.

#### Skepticism of CBOs

While no participant expressed complete mistrust of CBOs, some mentioned reasons for their skepticism. These included concerns about CBOs’ unresponsiveness to community needs and an insufficient number of CBOs to meet growing community demand:

*“I don’t know* [if I can trust CBOs] *because I’m still waiting on a follow-up. So*,* I don’t know. I don’t know if I can trust them for anything*,* not even a callback. So*,* I don’t know.” —*Black pregnant participant, less than 30 years old.

*“And so*,* there’s not enough community-based agencies here. They’re trying to get some together. There’s a medical building on Third Street. There’s dental – there’s X community clinic that’s being rebuilt and all. So*,* they’re progressing. But they’re nowhere near what could be*,* and people don’t have to go across town to get services if they can get them in this community.” —*Black family member, 45 years old and older.

Also, one participant stated that they didn’t fully trust CBOs because they had a general lack of familiarity and understanding of the organizations:

*“I really don’t know too much about them*,* so I don’t think I’ll trust them as much. I don’t know nothing really about them. So*,* I can’t trust them. If I don’t know nothing about you*,* I can’t put my trust in you.” —*Black family member, 45 years old and older.

### Perceptions of the health system’s involvement in PV

Given the extensive corpus of literature on mistrust in the health system, we asked participants about their perceptions of the health system’s involvement in PV to help understand trust in PV. Overall, participants had a positive outlook on the health system’s involvement in PV. This was based on *prior positive experiences with the health system*,* the health system’s role in providing resources and information*, and *meeting community needs.*

#### Positive experiences with the health system

Participants attributed their positive perceptions of the health system’s involvement in PV to their own positive experiences with the system:

*“I feel good about it* [health system’s involvement in PV]. *I mean*,* it’s part of wellness. And I gave birth at X hospital*,* and I had a great experience there*,* too. […] I feel good about it*,* definitely.” —*Latine postpartum participant, 30–44 years old.

#### Health system’s role in providing resources and information

Participants valued the health system’s involvement in PV, recognizing its role in providing vital information and resources:

*“Because they* [the health system] *have a different perspective*,* as well as knowledge. Both hospitals have different knowledge*,* and they can bring different ideas and different thoughts to the table. So*,* it’s good to have different resources and different people working together because everybody’s got different opinions and different ideas. So*,* it’s good that they both joined to help with the services of the Pop-Up.” —*Black pregnant participant, less than 30 years old.

A participant further noted that the health system’s involvement in PV acts as a critical resource-information bridge for the community, with hospitals and clinics serving as key sources of knowledge about sociomedical interventions like PV. The participant shared that she only learned about a CBO that supports Black pregnant people*—*and serves as a PV partnering organization*—*through one of her healthcare providers:

*“I feel good. I’m surprised a lot more major health providers aren’t a part of it. Because there are pregnant people that go to X hospital*,* even though it is a private company. But still*,* I’m surprised that you know*,* they’re*,* they’re not a part of it […] I would* [like more hospitals to be involved], *because that’s how you get the information out there. You know*,* that’s how you spread the word*,* that’s how you are more involved in your community*,* because had it not been for the intern that was my therapist at X hospital*,* I would have never known about Black Infant Health.” —*Black pregnant participant, between 30 and 44 years old.

#### Meeting community needs

Some participants supported the health system’s involvement in PV, noting the high demand for clinical services in their community and advocating for greater health system engagement to better meet these needs:

*“That’s good. We need more hospitals involved. We’ve got sick children. We’ve got lupus. We need all of our hospitals to be involved. Not just two or three. All of them. That’s how I feel […] People don’t just go to X and X hospital. They go to all these hospitals around San Francisco […] I think all the hospitals should be involved and let their patients know.” —*Black family member, 45 years old and older.

One participant noted that the health system’s involvement in PV demonstrates its recognition of the community’s need for clinical services and reflects its support for the community:

*“I think it’s pretty awesome that they’re coming together and noticing that these are the things that women need […] Why is it important for them to be involved? To let us know that there are*,* you know*,* health providers or doctors that are on our side and notice that this is something that we struggle majority of the time while being pregnant or after being pregnant. So having these services done by them*,* I guess*,* is a plus and a must.” —*Multiracial pregnant participant, between 30 and 44 years old.

#### Reservations about the health system’s involvement in PV

While most participants appreciated the health system’s involvement in PV, a few expressed concerns, emphasizing the importance of the system acknowledging and addressing its role in systemic issues. They also emphasized the importance of the health system understanding its position within the broader context of mistrust in the Black community.

“*I actually think that that is fine and good*,* as long as they recognize that they are part of the problem [laughs] and are working towards like becoming a part of a solution.” —*Black postpartum participant, 45 years old and above.

*“That’s the fact that they* [the health system] *are involved shows […] There is a caution*,* and I don’t want to speak for the whole Black community*,* but I know that I have and a lot of people I know who are Black*,* especially women when it comes to doctors and our trust to the system […]There’s a mistrust in this country*,* and I think it’s based and it’s valid […] when it comes to the medical field and how they have interacted with Black people in this country*.” *—*Black pregnant participant, between 30 and 44 years old.

## Discussion

We aimed to assess individuals’ perceptions of trust in PV and explore their trust perceptions of the health system and CBOs, as well as the health system’s involvement in PV. Trust in the PV model was generally high among participants and was influenced by factors such as race and ethnicity, language, history of preterm birth, and food insecurity, as found in the bivariate analyses. Qualitative findings revealed that trust in both the health system and CBOs was shaped by experiences of person-centered care and organizational familiarity. Trust in CBOs was additionally influenced by their emphasis on holistic care, relatability, and responsiveness to community needs. Conversely, distrust in the health system stemmed from experiences of racism and discrimination, inadequate preventative care, and the system’s neglect of Black women’s health. Participants held mixed views on the health system’s role in PV, with most highlighting its positive contributions, while a few voiced skepticism due to ongoing structural racism and inequities in care.

To the best of our knowledge, this study is the first to use the trust scale to assess trust in care from a community-institutional co-led care model. Although not directly comparable given the adaptations we made, the trust scores for PV are generally higher than scores obtained using the trust scale in healthcare settings [44]. The high levels of trust in PV are likely linked to PV’s cross-sector collaborative approach to care delivery that includes known trusted sources such as CBOs. Research also shows that such cross-sector partnerships promote knowledge sharing and support the development, integration, and dissemination of diverse information, resources, and care delivery approaches [49]. These collaborations often result in services that are more relevant, culturally affirming, and responsive to community needs [50], thereby fostering greater trust in the model.

We observed an inversion of the direction of association between race and ethnicity and trust in the bivariate and multivariate analyses, suggesting that confounding or interaction among covariates influenced the crude associations, which became clearer after adjustment. While the associations between race and ethnicity, language, history of preterm birth, food insecurity, and trust did not remain significant in the multivariate analysis—likely as a result of controlling for other covariates—they are meaningful and supported by qualitative data and prior research. First, because PV centers Black families, services are focused on Black families’ experiences, and initial challenges with translators affected the ability of non-English speaking participants to effectively navigate the Village [40]. This may have contributed to higher mistrust among Latine and Spanish-speaking individuals. These findings highlight the critical importance of addressing language barriers when assessing racial and ethnic disparities in PV.

Second, lower trust among individuals with a prior preterm birth is likely shaped by prior exposure and experiences [51, 52]. A history of preterm birth is a particularly traumatic experience due to the risk of severe infant injury or death [53] and has potentially profound and lasting effects on family well-being. Nearly half of mothers and a third of fathers experience post-traumatic stress disorder (PTSD) following a preterm birth [54]. For women of color, this is often compounded by negative healthcare experiences, including disrespectful care and unmet informational needs [55]. The cumulative impact of this trauma—of the preterm birth itself and systemic mistreatment—can deepen distrust toward the health system. Therefore, training providers to recognize and address trauma-related needs is crucial to prevent unintentional re-traumatization [56].

The association between food insecurity and trust may be understood through the lens of Maslow’s hierarchy of needs: individuals must first satisfy basic physiological and safety needs—such as food and housing—before pursuing higher-order needs such as belonging or self-actualization [57]. Thus, when these basic needs are unmet, attention and resources are often directed toward survival needs at the expense of being able or willing to engage with services perceived as peripheral to their unmet urgent needs. Although food was provided at PV events, this may not have been sufficient to meet the needs of individuals experiencing food insecurity. Consequently, if PV is not perceived as addressing these immediate needs, they might be less inclined to trust the model, a relationship that warrants further investigation.

That trust in the health system and CBOs is linked to person-centered care and service satisfaction is unsurprising. Transparent communication fosters shared decision-making, empowers individuals, and builds trust [58, 59]. Additionally, patient satisfaction—driven by provider responsiveness, clear explanations, and respectful interactions—is a key contributor to trust [60, 61]. Prior research shows that staff attitudes are the strongest predictor of satisfaction [62], and nearly half of Black pregnant women’s satisfaction with prenatal care is attributed to provider communication, including explanations, empowerment, and interpersonal style, such as friendliness and perceived discrimination [63]. The importance of person-centered care—characterized by respectful, open communication and shared decision-making—in fostering trust is therefore evident.

Trust in CBOs, unlike the health system, was also largely attributed to CBOs’ prioritization of holistic care, relatability, and commitment to addressing community needs. CBOs are often deeply rooted in the communities they serve, giving them a nuanced understanding of how social determinants of health—such as race and ethnicity, education, and income—interact to impact health and well-being [64]. This allows them to deliver services beyond clinical care and address structural inequities [65, 66]. Further, their holistic, personalized approach, grounded in lived experience, may foster trust. CBOs also often benefit from having staff and organizational leaders who share similar cultural and socioeconomic backgrounds with the communities they serve, creating mutual understanding and comfort; this relatability may enhance feelings of respect, belonging, safety, and trust [67]. Moreover, CBOs often serve as advocates for minoritized communities, advocating for resources, policy reforms, and social justice [68]. As a result, minoritized individuals may be more inclined to trust organizations that share their values, actively strive to meet their needs, and address structural inequities [30, 69].

The association between experiences of racism and discrimination and lower trust in the health system is well-documented in the literature [70]. This distrust stems from the complex interplay of individual, interpersonal, institutional, and structural racism. At the individual level, racist beliefs and ideologies can manifest as interpersonal racism, leading healthcare providers, influenced by implicit biases, to treat Black patients poorly, such as perceiving them as less compliant with medical directives [71] and dismissing their pain and other concerns [72]. These ingrained racist ideologies and practices are further reinforced at the institutional level, leading to significant disparities in care quality [55, 73, 74]. Ultimately, interpersonal and institutional racism converge to form structural racism, where racist ideas and beliefs are enshrined in laws and policies that allocate resources in ways that disempower and dehumanize historically minoritized populations [75, 76], perpetuating unequal access to high-quality care and poorer maternal and infant health outcomes. These all contribute to distrust of the healthcare system. While recent initiatives, such as implicit bias training, have aimed to reduce discrimination and build trust in the health system, they are insufficient alone. Addressing racial health inequities requires bold, innovative partnerships and coordinated advocacy among health systems, community-based organizations, and government agencies [77, 78]. These collaborations must go beyond surface-level interventions and focus on addressing the root causes of racial health inequities—structural racism—by advocating for essential national, local, and institutional policy changes [77, 78].

Given the complex history between minoritized populations and the health system, it was important to explore perceptions of the health system’s involvement in PV, as this has significant implications for building trust with these communities. Our findings revealed a duality in perspectives. Most participants viewed the health system’s involvement in PV positively, citing that it meets their needs. A possible explanation is that people are more likely to trust the health system when their health needs are met, thus a higher acceptance of the health system’s involvement in PV. However, we also found that a few individuals were skeptical of the health system’s involvement in PV, viewing it as complicit in perpetuating racial health inequities rather than a reliable partner in care. This skepticism and distrust may reflect broader patterns of structural inequities in healthcare, with roots in historical injustices such as slavery, during which the suffering and exploitation of enslaved women’s bodily autonomy for the sake of science inadvertently led to the establishment of the field of gynecology [79]. This legacy of medical racism continued with egregious ethical violations, such as the Havasupai Diabetes Project, where blood samples from the Havasupai Tribe were misused for unauthorized research [80], and the Tuskegee Syphilis Study, in which Black men were deliberately denied treatment to observe the natural progression of syphilis [81], contributing to profound distrust in the health system. To genuinely build trust with historically minoritized populations, the health system needs to proactively recognize and confront its own biases. This requires a multi-faceted approach: openly acknowledging the historical context of medical and structural racism, providing comprehensive training for staff that is both culturally affirming and trauma-informed, actively engaging with the community and partnering with CBOs, and establishing transparent practices that show a dedication to health equity [82].

Our findings suggest that authentic engagement in cross-sector, collaborative care models, such as PV, can help healthcare systems build and earn trust within the communities they serve. By partnering with CBOs to provide services, healthcare providers can adopt more person-centered approaches that reflect trusted community norms. This collaboration will also signal a commitment to deliver care in more community-centered and responsive ways. Given that trust is essential to achieving health equity, payers and public health departments should incentivize healthcare institutions and CBOs to form PV-like cross-sector collaborations that focus on trust-building. California’s state Medicaid program has made such efforts possible through its CalAIM Community Reinvestment policy, which requires Medicaid managed care plans to fund initiatives that address unmet needs and will have a significant impact on the local community’s health and well-being. Evaluations of such models should assess trust as a critical mediator of health-seeking behaviors and other outcomes.

### Strengths and limitations

This study has some limitations and strengths. First, since the evaluation took place within a real-world setting and the model’s dynamic co-creation and iteration approach enabled it to adjust to the needs expressed by people, the model’s consistency varied over time. Secondly, although our sample closely reflected the priority population, the use of purposive and convenience sampling methods limits the generalizability of our findings to broader populations. Nevertheless, the insights gained are likely relevant to similar communities and contexts. Third, no statistically significant predictors emerged in the final multivariate model, likely due to lack of statistical power, homogeneity of the sample, and measurement constraints, including potential reporting bias, limited ability to capture complex or non-linear relationships, and unmeasured confounded and contextual factors. Thus, the findings should be interpreted cautiously, and future studies should include more diverse, sufficiently powered samples. A significant strength of this study is the adaptation of a validated quantitative trust measure, enhancing validity and reliability. Additionally, this is one of the few studies to evaluate trust in a sociomedical intervention and the first study, to our knowledge, to explore individuals’ perceptions of the health system’s involvement in such an intervention.

## Conclusions

The pilot implementation of the Pregnancy Village model, the SF Family & Pregnancy Pop-Up Village, fostered high levels of trust among Black and other minoritized pregnant individuals and their families in San Francisco, California. These high trust perceptions suggest that cross-sector models like PV can bridge historical divides between community members and health systems by creating spaces where healthcare institutions can interact with, learn from and collaborate with community-based organizations in real time, and can be more community-driven, responsive, and structurally accountable than may be feasible in their usual settings. The high trust perceptions highlight the importance of sustained cross-sector collaborations between community and institutions. Future research should explore longitudinal outcomes of trust-building efforts, assess scalability across diverse settings, and examine the mediating role of trust in health outcomes. Policy efforts should prioritize sustained funding and institutional-community co-leadership to ensure models like PV thrive and evolve.

## Supplementary Information

Supplementary Material 1.

## Data Availability

The datasets generated and/or analyzed during the current study are not publicly available due to privacy and ethical restrictions but are available from the corresponding author upon reasonable request.
